# Expression of a Novel D4 Dopamine Receptor in the Lamprey Brain. Evolutionary Considerations about Dopamine Receptors

**DOI:** 10.3389/fnana.2015.00165

**Published:** 2016-01-06

**Authors:** Juan Pérez-Fernández, Manuel Megías, Manuel A. Pombal

**Affiliations:** Neurolam Group, Department of Functional Biology and Health Sciences, Faculty of Biology – Centro de Investigaciones Biomédicas – Instituto de Investigación Biomédica de Vigo, Uiversity of VigoVigo, Spain

**Keywords:** dopamine receptors, D4 receptor, lamprey, *in situ* hybridization, D2 class receptors, brain evolution

## Abstract

Numerous data reported in lampreys, which belong to the phylogenetically oldest branch of vertebrates, show that the dopaminergic system was already well developed at the dawn of vertebrate evolution. The expression of dopamine in the lamprey brain is well conserved when compared to other vertebrates, and this is also true for the D2 receptor. Additionally, the key role of dopamine in the striatum, modulating the excitability in the direct and indirect pathways through the D1 and D2 receptors, has also been recently reported in these animals. The moment of divergence regarding the two whole genome duplications occurred in vertebrates suggests that additional receptors, apart from the D1 and D2 previously reported, could be present in lampreys. We used *in situ* hybridization to characterize the expression of a novel dopamine receptor, which we have identified as a D4 receptor according to the phylogenetic analysis. The D4 receptor shows in the sea lamprey a more restricted expression pattern than the D2 subtype, as reported in mammals. Its main expression areas are the striatum, lateral and ventral pallial sectors, several hypothalamic regions, habenula, and mesencephalic and rhombencephalic motoneurons. Some expression areas are well conserved through vertebrate evolution, as is the case of the striatum or the habenula, but the controversies regarding the D4 receptor expression in other vertebrates hampers for a complete comparison, especially in rhombencephalic regions. Our results further support that the dopaminergic system in vertebrates is well conserved and suggest that at least some functions of the D4 receptor were already present before the divergence of lampreys.

## Introduction

Dopamine, a neurotransmitter with a broad influence in the central nervous system of vertebrates, has been suggested to be involved in numerous and important functions including locomotion, learning, feeding, and reward behavior (Missale et al., 1998; Beaulieu and Gainetdinov, 2011). Its role is reflected in the fact that severe diseases, such as Parkinson’s disease or Tourette’s syndrome, are caused by alterations in the dopaminergic system (Beaulieu and Gainetdinov, 2011). Dopamine acts through two evolutionarily convergent classes of G-protein-coupled receptors (GPCRs), D1 and D2 (Kebabian and Calne, 1979; Beaulieu and Gainetdinov, 2011). In the last decades, several reports suggest that the dopaminergic system is highly conserved in vertebrates, according to the data reported in lampreys (Pierre et al., 1994, 1997; Pombal et al., 1997; Pierre-Simons et al., 2002; Abalo et al., 2005; Barreiro-Iglesias et al., 2010; Robertson et al., 2012; Ericsson et al., 2013; Pérez-Fernández et al., 2014b). These animals, together with hagfishes, represent the oldest living branch of vertebrates that diverged from the main branch leading to vertebrates around 560 million years ago (Kumar and Hedges, 1998).

The lamprey dopamine expression pattern (Pierre et al., 1994, 1997; Pombal et al., 1997; Pierre-Simons et al., 2002; Abalo et al., 2005) is quite similar to that reported in other vertebrates. It has recently been shown that dopamine has a conservative role in modulating the excitability of D1 and D2 receptor expressing striatal neurons of the direct and indirect pathways (Robertson et al., 2012; Ericsson et al., 2013). Moreover, the expression pattern of the D2 receptor in the river lamprey shows striking similarities to that in other vertebrates and its anatomical profile suggests that many of its functions are highly conserved in vertebrates (Pérez-Fernández et al., 2014b). A critical period in the evolution of D1 and D2 dopamine receptors, as well as of other gene families, is thought to have occurred during the early evolution of vertebrates (500–800 million years ago; Vandepoele et al., 2004), when two whole genome duplications (WGD) proposed by the 2R hypothesis are thought to have taken place (Ohno, 1970; Panopoulou and Poustka, 2005; Caputo et al., 2013). These two rounds of WGD increased the number and variance of genes, and the repertoire of dopamine receptors present nowadays in a lineage is therefore a consequence of gene duplications plus gene losses that occurred during its evolutionary history. A third WGD round (3R) occurred in the teleost lineage (reviewed in Meyer and Van de Peer, 2005). Although both classes of dopamine receptors are present in all species of vertebrates studied so far, there are different members depending on the lineage. Mammals possess D1A and D1B receptors belonging to the D1 class, whereas chondrichthyans, amphibians, and turtles have an additional receptor known as D1C (Callier et al., 2003). Lepidosaurs show a D1D receptor, nowadays considered an orthologous receptor of the D1C subtype (Yamamoto et al., 2013). In teleosts, two different D1A receptors (D1A1 and D1A2) have been found (reviewed in Le Crom et al., 2003), in agreement with the additional duplication occurring in this lineage. Within the D2 class, D2, D3, and D4 subtypes are present in most vertebrates studied so far (Kubikova et al., 2010). In teleosts, there are additional copies of the D2 and D4 receptors (Pasqualini et al., 2009) but only a single copy of the D3 receptor has been found. Concerning agnathans, numerous data suggest that this group branched from the common vertebrate tree at least after the first WGD proposed by the 2R hypothesis (Kasahara, 2007; Osório and Rétaux, 2008; Caputo et al., 2013; Pérez-Fernández et al., 2013, 2014a; Smith et al., 2013), and therefore additional dopamine receptors could be present apart from the D1 and the D2 receptors already reported (Robertson et al., 2012; Pérez-Fernández, 2013). Given the high degree of conservation of the dopaminergic system, it is interesting to see if additional dopamine receptors are present in lampreys in order to shed light into the evolution of this important modulatory system.

Most studies available on the dopaminergic system are focused on the D1 and the D2 receptors, whereas few concern the other subtypes. Although the D4 receptor was found long time ago, just after the discovery of cloning tools (Van Tol et al., 1991), only a few studies exist analyzing its anatomical profile (see below). Moreover, important discrepancies exist among these studies so that a detailed expression pattern of the D4 receptor is not clear yet, even in mammals. The D4 receptor has attracted interest because of its potential role mediating the effects of some atypical antipsychotics or drug addiction (Rondou et al., 2010; Sampson et al., 2014), but historically, it has been difficult to study because of the lack of true selective agonists (see Rondou et al., 2010) and studies to identify specific ligands against the D4 receptor are still being carried out nowadays (Abdelfattah et al., 2013; Kügler et al., 2013; Lacivita et al., 2014; Leopoldo et al., 2014; Sampson et al., 2014). In addition, the antibodies developed against the D4 receptor have been shown to be insufficiently selective and sensitive (Kubikova et al., 2010; Rondou et al., 2010), and only a few studies have used *in situ* hybridization to analyze the D4 receptor expression pattern (Meador-Woodruff et al., 1994, 1996, 1997; Lidow et al., 1998; Callier et al., 2003; Noaín et al., 2006; Boehmler et al., 2007; Zhu et al., 2007; Kubikova et al., 2010). Altogether, this makes that the expression pattern of this receptor is still a matter of debate (Leopoldo et al., 2014).

In this study, we characterized the anatomical expression of the D4 receptor in the sea lamprey brain by using *in situ* hybridization and compared it with that of other vertebrates from an evolutionary point of view. The comparison of brain compartments between different vertebrate species was based on the neuromeric model of the vertebrate brain, which is of help to establish homologies (Pombal and Puelles, 1999; Pombal et al., 2001; Puelles and Rubenstein, 2003; Pombal et al., 2009; Martínez-de-la-Torre et al., 2011; Pombal and Megías, 2011).

## Materials and Methods

### Dopamine D4 Receptor Sequence

Dopamine receptor sequences were searched by using the BLAT Search Genome from the University of California, Santa Cruz (UCSC) Genome Bioinformatics site^1^, which in turn is based on data produced by The Genome Institute at Washington University School of Medicine in St. Louis (Kent, 2002; Kent et al., 2002; Meyer et al., 2013). The different vertebrate dopamine receptor sequences used as queries were obtained from GenBank^2^. Additional searches were performed with the trace archives BLAST tool, by using the whole genome shotgun option, and the Ensembl project lamprey data^3^.

Sequence comparisons were carried out with the Basic Local Alignment Search Tool (BLAST^4^) by NCBI, and the CLC Sequence Viewer 6.4 (CLC bio A/S, Germany). Alignments shown in the “Results” section were obtained with this last program. Transmembrane domains were uncovered by using SMART (simple modular architecture research tool^5^; Letunic et al., 2009), and the OCTOPUS tool (prediction of membrane protein topology and signal peptides^6^). For the prediction of exons and introns, the GENSCAN Web Server at MIT was used (Burge and Karlin, 1997^7^).

### Phylogenetic Analysis

The phylogenetic tree was constructed by the alignment of representative amino-acidic sequences of D2 class dopamine receptors of other vertebrates by using the ClustalW tool of MEGA 5.0 (Tamura et al., 2011). The conditions were pairwise alignment gap opening penalty 30, pairwise alignment gap extension penalty 0.75, multiple alignment gap opening penalty 15, multiple alignment gap extension penalty 0.3 using the Gonnet protein weight matrix. The method used for phylogenetic analysis was neighbor-joining with Poisson-corrected distances, complete deletion of gaps and 1000 bootstrap replicates. Final alignment and phylogenetic conditions were chosen based in a visual checking of the alignments and consistency of the tree topology with the evolutionary history of dopamine receptors and the species included in the analysis.

### Expression Pattern

The *in situ* hybridization studies were performed on 4 adults of sea lamprey *Petromyzon marinus*. Animals were obtained from a local commercial supplier. The experiments were carried out following the guidelines on animal care and experimentation established by the Spanish Royal Decree 223/1988 and the revised Royal Decree 1021/2005, and by the local animal welfare ethical committee of the University of Vigo. Lampreys were deeply anesthetized in 0.1% tricaine methanesulfonate (MS-222, Sigma) and killed by decapitation. The brains and rostral spinal cord were quickly dissected out and fixed overnight with 4% paraformaldehyde in buffer phosphate 0.1 M, pH 7.4 (PB). Then, they were cryoprotected in 30% sucrose in PB, embedded in OCT compound (Tissue-Tek, Sakura, Torrance, CA, USA), frozen at –80°C, and 20 μm thick sections were obtained in a cryostat (Microm HM505 E). Sections were collected on Superfrost slides (Menzel GmbH & Co. Germany), and immediately used for *in situ* hybridization or stored in a –80°C freezer until use.

The probe was obtained as follows. The partial D4 sequence corresponding to the final portion of the protein was synthesized (GenScript, Hong Kong) and subcloned into the pGEM-T easy vector (Promega, Madison, WI, USA). Subsequently, PCRs were performed with primers designed against the SP6 and T7 sequences flanking the insert. The probe was synthesized from the PCR product by *in vitro* transcription using SP6/T7 RNA polymerases (Roche, Germany) and digoxigenin-UTP (Roche, Germany), according to the manufacturer’s instructions.

Sections were processed for *in situ* hybridization. Briefly, sections were directly washed three times for 10 min in PBS, and then acetylated for 5 min. Subsequently, they were washed three times for 10 min in PBS and incubated in a prehybridization solution for 2 h containing 50% formamide, 5X SSC pH 7.0, 5x Denhardts’, 500 μg/ml salmon sperm DNA and 250 μg/ml yeast RNA. Hybridization process was carried out in a humid chamber at 60°C overnight adding the probe to a final concentration of 500 ng/ml. Subsequently, several astringent washes with SSC and formamide in different concentrations were performed to stop the process (2 x 15 min in 1X SSC at 60°C, 0.5X SSC in formamide for 1 h at 60°C, 1X SSC 15 min at 60°C), followed by an RNAse A treatment (RNAse A to a final concentration of 20 μg/ml, 30 min at 37°C) to remove the unbound and non-specific linked probes. Then, sections were rinsed (2 × 10 min in 1X SSC at 60°C), washed with MABT (3 × 5 min) and blocked in MABT containing 10% HINGS (Heat inactivated goat serum) for 1 h. Finally, they were incubated overnight with anti-digoxigenin Fab-fragments conjugated with alkaline phosphatase diluted 1:1500 in MABT containing 0.5% HINGS at 4°C. Then, sections were washed in MABT (4 × 20 min), preincubated in staining buffer (2 × 45 min; Trizma 0.1 M, pH 9.5, NaCl 0.01 M, levamisole 0.25 mg/ml) and incubated overnight in darkness in staining buffer containing 20 μl/ml NBT-BCIP substrate. The phosphatase alkaline reaction was stopped by washes in MABT. Finally, sections were dehydrated and coverslipped. Adjacent series were incubated with the sense probe to be used as a control, and no labeling was observed.

## Results

### Molecular Structure and Phylogeny

**FIGURE 1:**
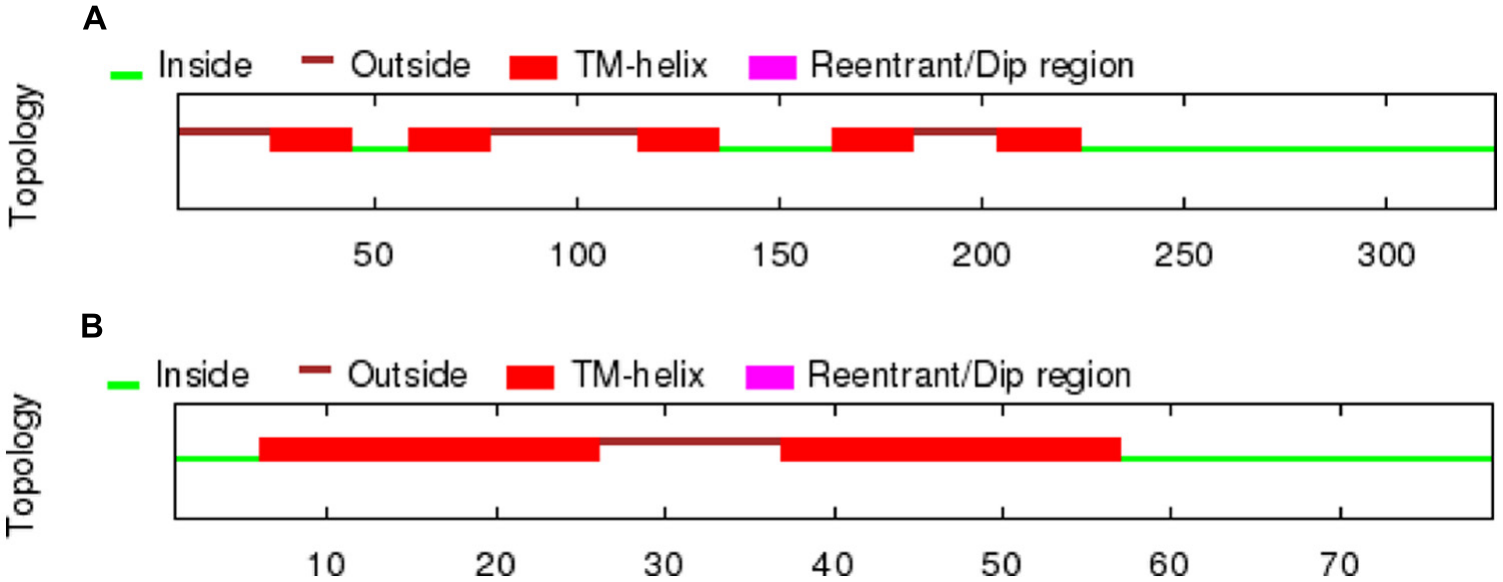
**TM regions deduced by OCTOPUS for the lamprey D4 receptor sequences retrieved from the UCSC Genome bioinformatics website (A) and the trace archives of the NCBI (B)**.

A partial sequence similar to the D4 receptor was retrieved from *P. marinus* contig 32392 by using the lamprey BLAT search (UCSC Genome bioinformatics). The whole sequence could not be obtained due to a gap present in the contig. A bigger fragment of the same region was found in the contig 476906, although the whole sequence was not present because it was interrupted by the end of the contig. The obtained sequence exhibited introns in its primary transcription region and therefore the Genscan program, which allows the prediction of the gene sequence by differentiating exons and introns, was used to predict the coding region. As expected, the sequence of the protein obtained was not complete and its final portion is not included in this fragment. The obtained protein sequence spanned five transmembrane (TM) domains as deduced by OCTOPUS program (**Figure 1**), which are likely to be the five first ones according to the Genscan program. The first methionine start codon as deduced by Genscan was also placed in a strong consensus motif for a ribosomal binding site, with a cytosine in position -1 and an adenine in position -4 (data not shown, Kozak, 1996). The alignments with other D4 sequences also showed that this fragment corresponds to the first five TM regions and it spanned a fragment of the third extracellular loop as well. This region was also retrieved from the Ensembl project server (Ensembl Protein: ENSPMAP00000010574).

**FIGURE 2:**
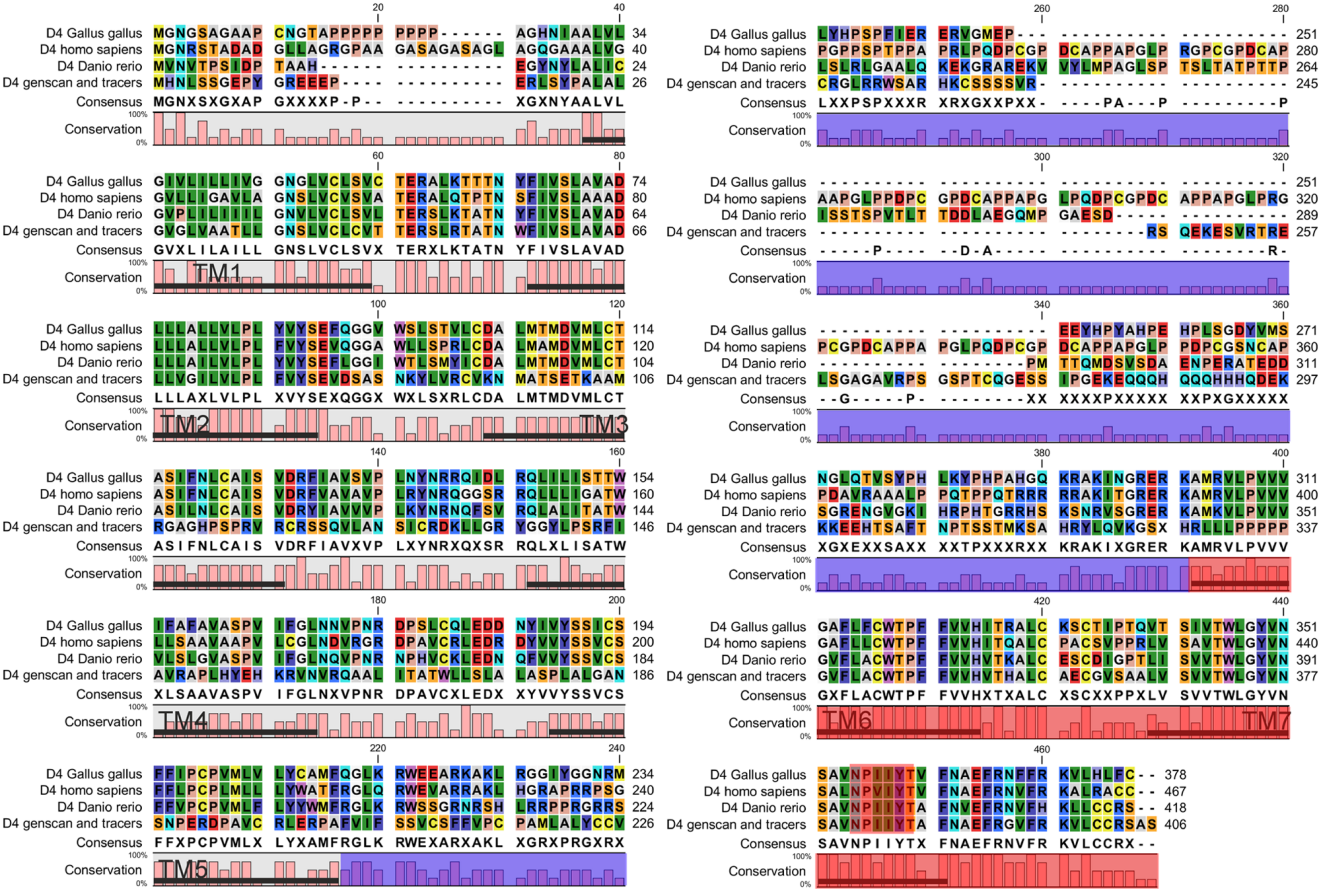
**Alignment of the D4 sequence of lampreys with the D4 receptor sequences of other vertebrates.** The area shaded in blue corresponds to the third intracellular loop where a fragment of the D4 sequence of lampreys is missing. The region used to design the probe for the *in situ* hybridization is shaded in red. The NPxxY(x)(5,6) motif, which connects TM7 to the last cytosolic helix in GPCRs, is shaded in red.

In order to retrieve the final portion of the protein, additional searches were carried out by using the NCBI trace archive databases. Although some additional fragments corresponding to the end of the D4 sequence were obtained, they did not overlap with the final part of the sequence obtained from the UCSC contig. Therefore, this implies that at least a portion of the D4 receptor sequence is missing in both the UCSC contig and the NCBI trace archives. These fragments were then searched again in the UCSC Genome bioinformatics database, and the final part of the protein was found in the contig 480875. The fragments retrieved from the trace archives were likely to be the last part of the protein because the OCTOPUS program deduced two TMs (**Figure 1**), and the alignments showed strongly conserved motifs in the last transmembrane regions, e.g., the NPxxY(x)(5,6) motif, which connects TM7 to the last cytosolic helix in GPCRs (Fritze et al., 2003; shaded in red in **Figure 2**). This region spanning the last TM regions and the end of the protein was used for the *in situ* hybridization probe construction (shaded in red in **Figure 2**). Altogether, it is likely that only a small fragment belonging to the third long extracellular loop is missing in the D4 sequence shown in **Figure 2**.

**FIGURE 3:**
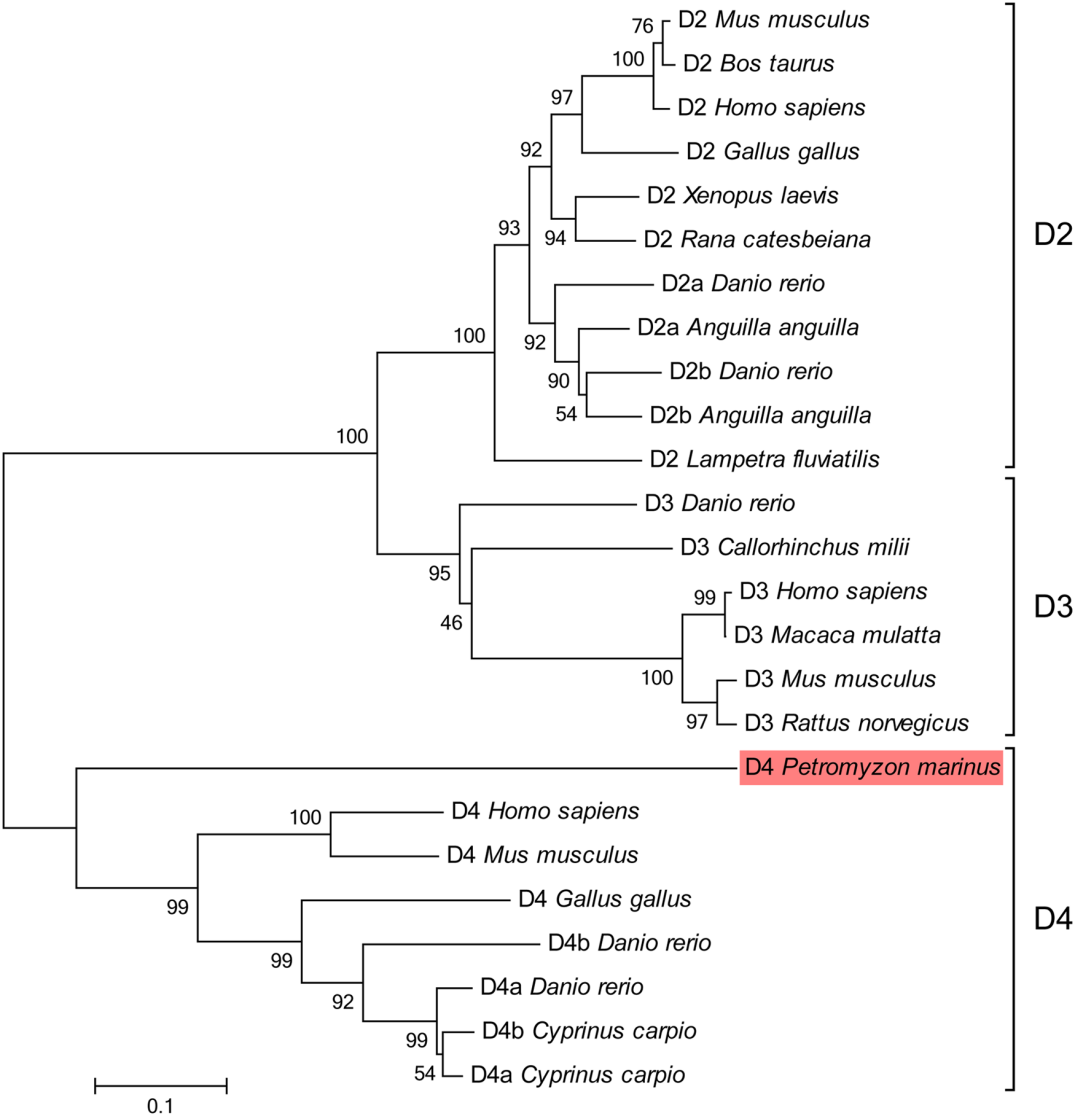
**Phylogenetic tree for the D2 class receptors.** The sequence of lamprey analyzed in the present study is shaded in red. GenBank accession numbers of the sequences included in the tree are: *Gallus gallus* D2, NP_001106761; *Homo sapiens* D2, EAW67225; *Rana catesbeiana* D2, BAI70438; *Bos taurus* D2, CAA35970; *Lampetra fluviatilis* D2, ADO23655; *Mus musculus* D2, NP_034207; *Xenopus laevis* D2, NP_001095212; *Anguilla anguilla* D2a, ABH06893; *Anguilla anguilla* D2b, ABH06894; *Danio rerio* D2a, NP_898891; *Danio rerio* D2b, NP_922918; *Danio rerio* D3, NP_898890; *Callorhinchus milii* D3, NP_001279680; *Homo sapiens* D3, P35462; *Macaca mulatta* D3, NP_001276826; *Mus musculus* D3, NP_031903; *Rattus norvegicus* D3, NP_058836; *Gallus gallus* D4, NP_001136321; *Mus musculus* D4, NP_031904; *Homo sapiens* D4, NP_000788; *Cyprinus carpio* D4a, CAA74974; *Cyprinus carpio* D4b, CAA74977; *Danio rerio* D4a, NP_001012634; *Danio rerio* D4b, NP_001012636. The percentages of replicate trees in which the associated taxa clustered together in the bootstrap test (1000 replicates) are shown next to the branches. The tree is drawn to scale, with branch lengths in the same units as those of the evolutionary distances used to infer the phylogenetic tree. The scale bar refers to a phylogenetic distance of 0.1 amino acid substitutions per site. See Section “Materials and Methods” for alignment and tree construction conditions.

The phylogenetic analysis supported that the sequence we found is a D4 receptor subtype, because it clustered basal to other D4 sequences with high bootstrap values (**Figure 3**). The blast analysis of the fragments retrieved from the NCBI trace archives also confirmed their relationship with the D4 receptors reported for other vertebrates (data not shown).

### Expression Pattern

**FIGURE 4:**
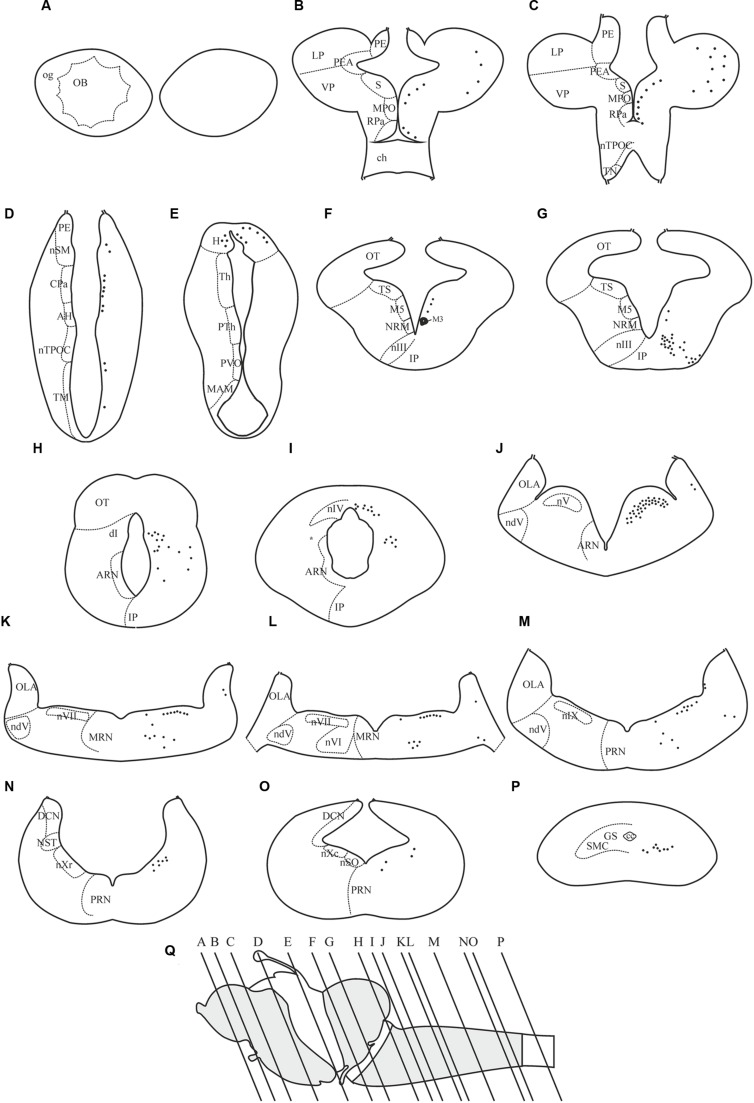
**Distribution of positive neurons for the D4 receptor in representative transverse sections of *P. marinus* brain from rostral to caudal (A–P)**. The location of the different areas and nuclei are represented in the left side, whereas the location of the D4 positive cells is indicated by black spots in the right side of each drawing. The rostrocaudal level of each section is shown in a schematic drawing of a lateral view of the sea lamprey brain **(Q)**. For abbreviations, see list.

**FIGURE 5:**
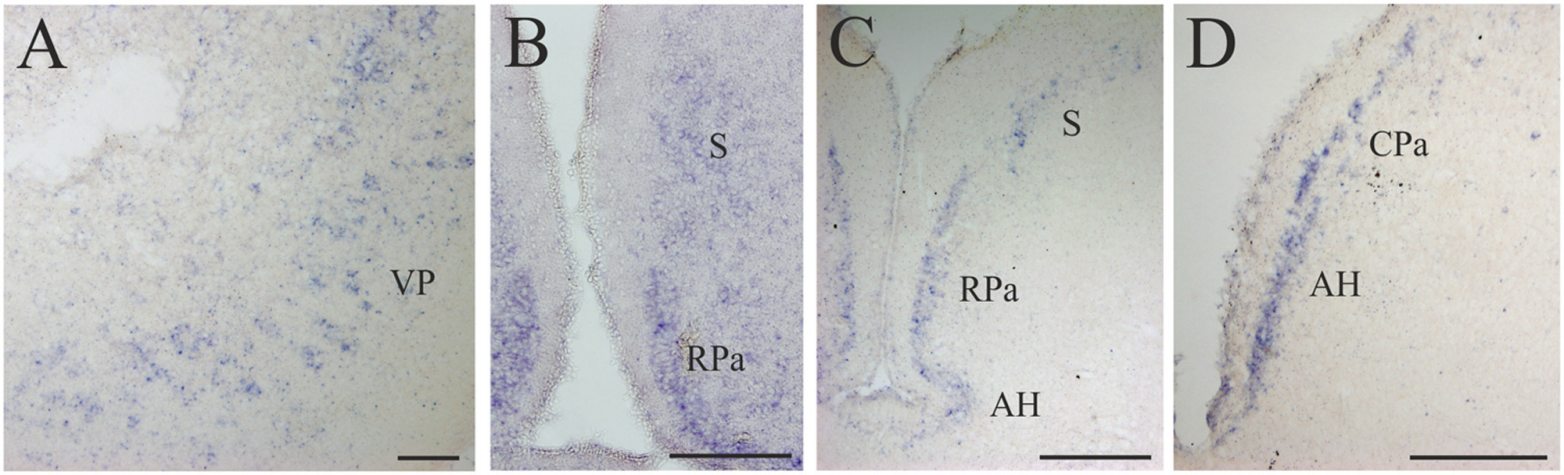
**D4 expression in the secondary prosencephalon. (A)** Transverse section showing the D4 positive cells of the VP. **(B)** Microphotograph showing D4 labeled cells in the RPa and the S. **(C)** Section caudally to that shown in B illustrating the expression of the D4 probe in the same two nuclei as well as in the AH. **(D)** Numerous D4 labeled cells are observed in the AH and in the CPa. For abbreviations, see list. Scale bar = 125 μm in **(A**,**B**,**D)**; 100 μm in **(C)**.

**FIGURE 6:**
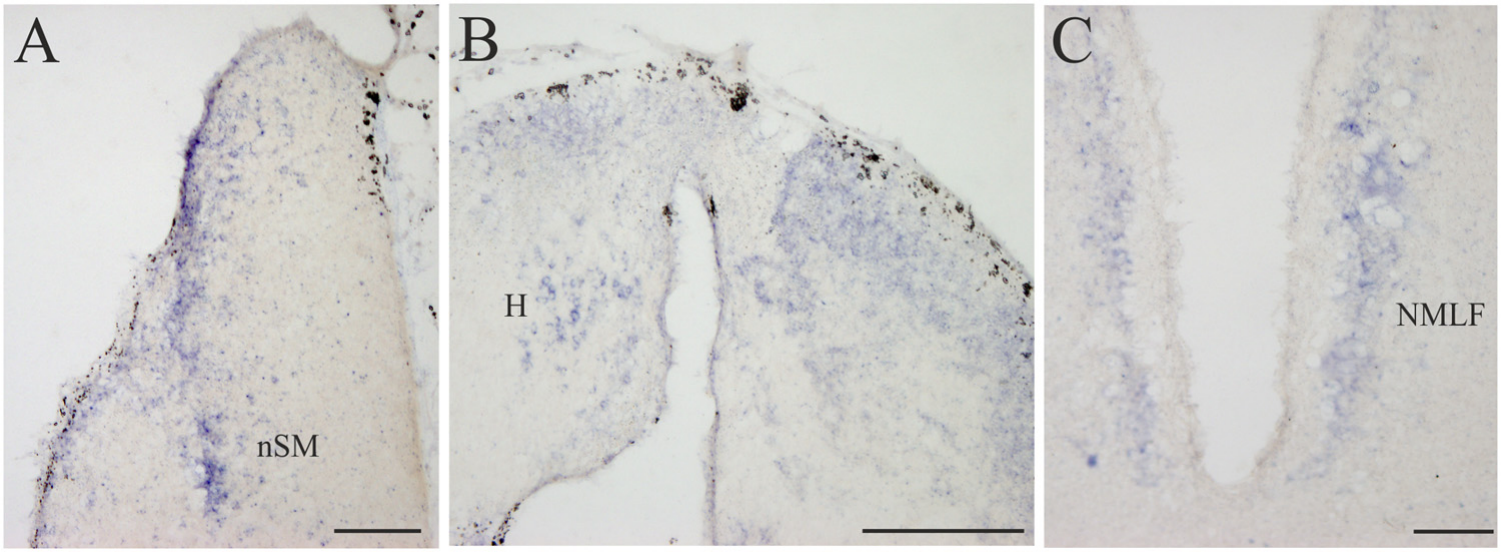
**D4 expression in the diencephalon. (A)** Several D4 labeled cells are observed in the prethalamic nSM. **(B)** Numerous and small D4 labeled cells are observed in the two portions of the sea lamprey H. **(C)** Some D4 positive cells are present in the NMLF. For abbreviations, see list. Scale bar = 200 μm in **(A,C)**; 100 μm in **(B)**.

**FIGURE 7:**
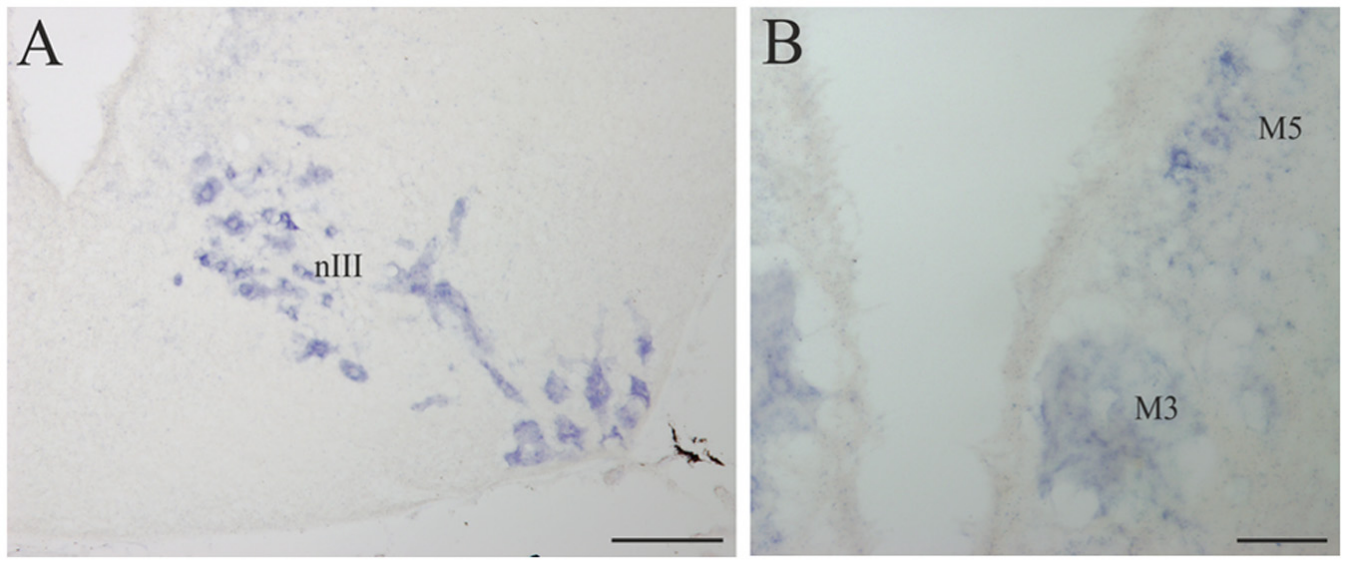
**D4 expression in the mesencephalon. (A)** Numerous and intensely stained cells are observed in the nIII, in the basal portion of the mesencephalon. **(B)** Transverse section through the mesencephalon at the level of the third Müller (M3) cell showing its D4 labeling as well as that of some cells in the M5 nucleus of Schöber. For abbreviations, see list. Scale bar = 200 μm in **(A)** 100 μm in **(B)**.

**FIGURE 8:**
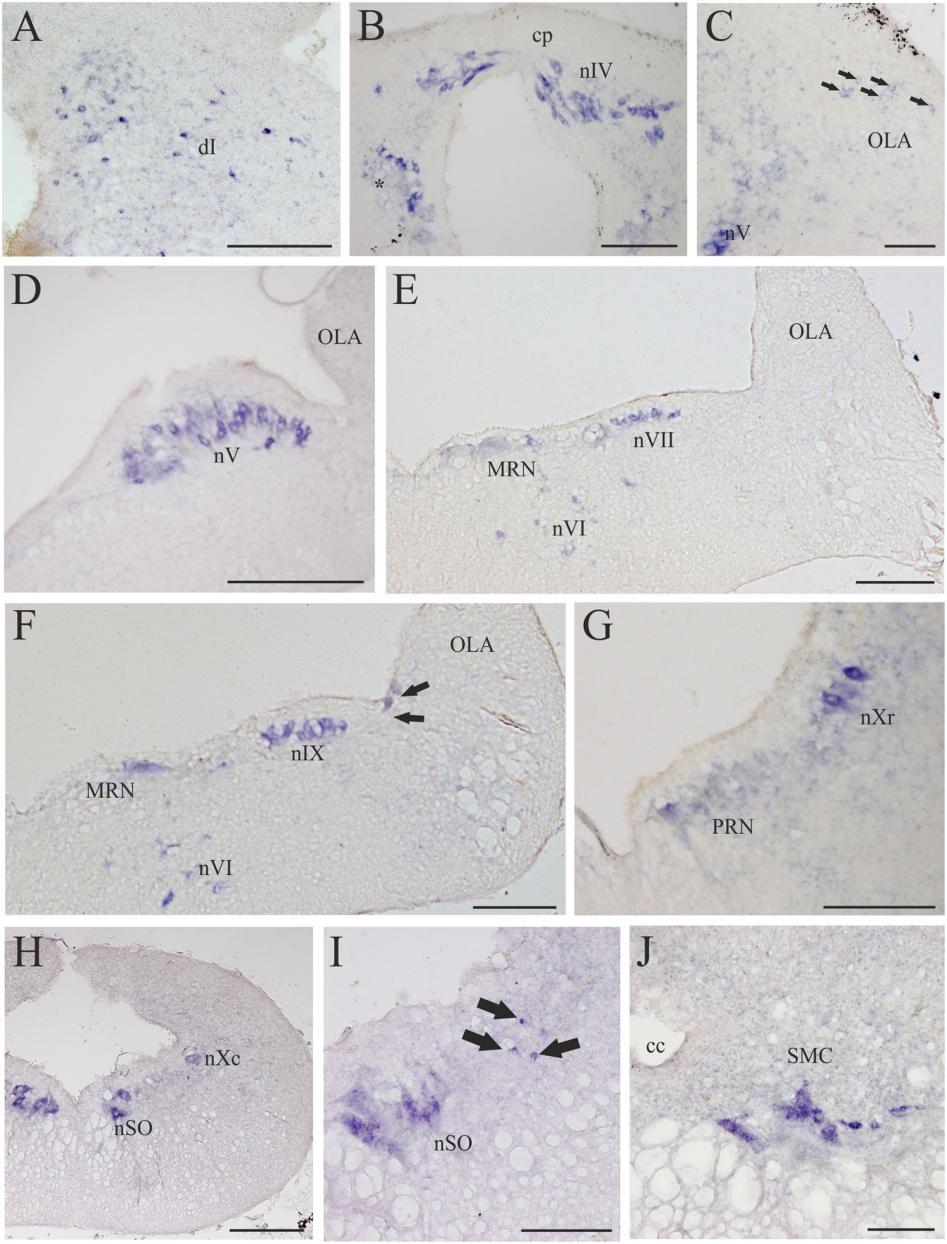
**D4 expression in the rhombencephalon and spinal cord. (A)** Numerous and scattered D4 positive cells in the dorsal isthmus. **(B)** Photomicrograph showing the strongly D4 labeled cells of the nIV, as well as ventrally to this nucleus (just close to the level of the first isthmic Müller cell; asterisk). **(C)** Section showing some weakly labeled D4 cells (arrows) in the rostral OLA, as well as other labeled cells in the rostral part of the nV. **(D)** Numerous and large D4 labeled motoneurons can be observed in the nV. **(E)** Transverse section showing D4 positive cells in the nVI and nVII. Some D4 labeled reticular cells are also present in the medial part of the reticular formation (MRN). **(F)** Numerous D4 positive cells are observed in both the nVI and nIX. Some reticular cells are also labeled. The black arrows point to two sensory cells of the primary medullary nucleus of the trigeminal nerve, which also express the D4 receptor. **(G)** Photomicrograph showing D4 labeled cells in the nXr, as well as in the PRN of the reticular formation. **(H)** Section showing D4 positive cells in the nXc, as well as in the nSO. **(I)** Intense labeled D4 cells are present in the nSO. The black arrows point to small D4 labeled cells located dorsolaterally to the motor nucleus. **(J)** Transverse section through the rostral spinal cord showing intensely labeled D4 cells in the SMC. According to the size and position they were identified as motoneurons. For abbreviations, see list. Scale bar = 250 μm in **(A,B,G,I)**; 100 μm in **(C,J)**; 500 μm in **(D,E,F,H)**; 125 μm in **(F)**.

The dopamine D4 receptor showed a restricted expression pattern in the sea lamprey brain (see **Figures 4**–**8**). The largest number of positive cells for the D4 receptor was found in the forebrain (see secondary prosencephalon below), but they generally show a weak labeling. However, compared to other parts of the brain, the telencephalon showed the scarcest expression for this receptor. In the mesencephalon, most of the D4 positive cells detected corresponded to motoneurons. In the rhombencephalon (hindbrain), as occurred in the mesencephalon, D4 positive cells were almost restricted to the motor nuclei of the cranial nerves (including both the somatomotor and the visceromotor nuclei), which were intensely labeled. The expression of D4 receptor is shown in schematic transverse sections from rostral (A) to caudal (P) in **Figure 4**.

#### Secondary Prosencephalon

Some D4 positive cells were present in the lateral (LP) and ventral (VP) pallial sectors of the evaginated telencephalic hemispheres (**Figures 4B,C** and **5A**). In the subpallium, a few D4 positive cells were detected in the S, representing a subpopulation within this nucleus. These cells were scattered and distributed along the striatal cell layer that is separated from the ventricle by a relatively thick neuropil (**Figures 4B,C** and **5B,C**). The most conspicuous D4 labeled cell population in the secondary prosencephalon was located in the RPa (**Figures 4B,C** and **5B,C**). Most cells of this nucleus expressed the D4 receptor and were grouped in a cell plate close to the ventricular surface, part of which bordered the preoptic recess. We could not clearly distinguish whether some of these cells belong to the epichiasmatic or suprachiasmatic nuclei. Although the RPa neurons were more strongly labeled than the other positive cells within the secondary prosencephalon, the labeling intensity was much weaker than that found in the mesencephalic and rhombencephalic cranial motor nuclei (compare **Figures 5B,C**, **7A,B** and **8A–J**). The D4 positive cells of RPa were caudally continuous with others labeled in the CPa (**Figures 4D** and **5D**). In addition, some D4 positive cells were also observed in the AH (**Figures 4D** and **5C,D**). Concerning the basal portion of the secondary prosencephalon, some D4 positive cells were located in the nTPOC, and were also scattered through the TN and TM (**Figures 4C,D**).

#### Diencephalon

All the D4 expressing cells found in the diencephalon of the sea lamprey were weakly labeled and distributed in a few cell populations. Some D4 positive cells were present in a subpopulation of the PE, which corresponds to the nSM (**Figures 4D** and **6A**). They constituted a medial band of cells that follows the trajectory of the stria medullaris toward the H as it courses through the PE. The most conspicuous labeled diencephalic structure was the H, which showed numerous small D4 labeled cells in both the left and right portions (**Figures 4E and 6B**). Finally, in the basal region of the first prosomere (p1), a weak D4 expression was found in some cells of the NMLF, including the two pairs of giant Müller cells (known as M1 and M2) present in this area (**Figure 6C**).

#### Mesencephalon

A strong D4 expression was detected in the mesencephalic basal region, which essentially corresponded to the motoneurons of the nIII (**Figures 4G** and **7A**). These motoneurons were homogenously labeled and distributed in a radial band extending from a periventricular position to the lateral surface of the brain, where their axons enter the third cranial nerve. They constitute the most rostral part of the somatic motor column and in these animals innervate three extraocular muscles, i.e., the rostral and dorsal rectus and the rostral oblique (Fritzsch et al., 1990). The intensity of the staining in the oculomotor cells, similarly as in other motoneurons (see below), is one of the strongest in the whole brain of the sea lamprey. The mesencephalic pair of giant M3 was also positive for the D4 probe (**Figures 4F** and **7B**). In addition, some weakly D4 labeled cells were also present in the region of the M5 nucleus of Schöber (**Figures 4F** and **7B**).

#### Rhombencephalon

Numerous D4 positive cells were detected in the rhombencephalon of the sea lamprey. In the rostralmost part of the isthmic region a numerous population of small D4 labeled cells was observed in a dorsal position (dI; **Figures 4H** and **8A**). Most of these cells were grouped in a medial location but others were laterally displaced and scattered through the tegmentum. Slightly caudal to this cell population, numerous D4 positive cells were detected in the nIV (**Figures 4I** and **8B**), which innervates the caudal oblique extraocular muscle (Fritzsch and Sonntag, 1988). In contrast to the general rule for vertebrates, in lampreys the nIV is dorsally located. In fact, some of its cells distributed alongside the so-called cerebellar commissure and were relatively large and elongate in shape, whereas those located more laterally were smaller and had round or slightly ovoid perikarya. All of them showed a quite strong labeling, similar to that observed in the mesencephalic nIII. Ventrally and ventrocaudally to the nIV, there were several strongly labeled D4 positive cells close to the ventricle, just at the level of the isthmic (I1) and pre-trigeminal (I2) large Müller cells.

The most intense D4 labeling was observed in motoneurons of the cranial nerves, including the nV, nVI, nVII, nIX, nX, and nSO. The most conspicuous of these nuclei is the nV, where the size and the shape of the D4 positive cells clearly indicated that they are motoneurons (nV; **Figures 4J** and **8C,D**). Caudally from this nucleus, and inside the same (visceromotor) column, were distributed the D4 positive motoneurons belonging to the nVII (**Figures 4K,L** and **8E**), nIX (**Figures 4M** and **8F**), and nX (**Figures 4N,O** and **8G,H**) motor nuclei; all these cells were smaller than those of the nV but were also intensely labeled. At the rostrocaudal level of the nVII and nIX but located more medially (topologically ventral), several intensely labeled D4 positive cells were identified in the nVI (**Figures 4L** and **8E,F**), which is the representative of the somatomotor column at this level. Inside the same column, but in the caudal rhombencephalon, there were numerous D4 positive neurons belonging to the nSO (**Figures 4O** and **8H,I**).

Some weakly to moderate labeled D4 cells were also found in the rhombencephalic reticular formation. Most of these labeled cells were located in the medial nucleus of the reticular formation (MRN; **Figures 4K,L** and **8E,F**), where large reticular cells are present. In the ARN no D4 positive cells were found, whereas only a few and weakly labeled cells were detected in the posterior reticular formation (PRN; **Figures 4M–O** and **8G**). Finally, a few cells located between the somatomotor and the visceromotor columns also exhibited D4 staining (arrows in **Figure 8I**). They were quite small but strongly labeled, being more numerous at the level of the nSO.

In the alar region of the rhombencephalon proper, some D4 positive cells were found inside the ventral nucleus of the OLA (**Figures 4J–M** and **8C**); they were moderately labeled and somewhat more numerous around the level of the rostral nV. At more caudal levels a number of subependymal D4 labeled cells were located just dorsally and close to the level of the sulcus limitans of His (**Figure 4M**, arrows in **Figure 8F**). They were moderately but consistently labeled throughout the rhombencephalon. These lamprey sensory cells, which in transverse sections of the brain are readily identified due to their size and particular location, were named “primary medullary and spinal nucleus of the trigeminal nerve” (PMSV) by Anadón et al. (1989), after labeling them by tracer application to the trigeminal nerve.

#### Spinal Cord

A strong labeling for the D4 receptor was found in the rostral portion of the sea lamprey spinal cord. Intense D4 positive cells that clearly correspond to spinal motoneurons were observed in the motor column of the gray substance (SMC; **Figures 4P** and **8J**).

## Discussion

The present study reports the expression of a new dopamine receptor in lampreys, the D4 receptor. Our results further support that the dopaminergic system was already well developed at the dawn of vertebrates. Our data are relevant since studies concerning the expression of the D4 receptor in mammalian species are very scarce in the literature, being almost non-existent in non-mammalian species.

### Expression Pattern of the D4 Receptor

The D4 receptor shows a restricted expression pattern in the sea lamprey brain, especially when compared to that of the D2 receptor (Pérez-Fernández et al., 2014b). D4 positive neurons were mainly found in the rhombencephalon, where many of the labeled cells corresponded to motoneurons of the different motor nuclei (including both somatomotor and branchiomotor nuclei). It is difficult to precisely compare our results in the sea lamprey with studies in other vertebrates for several reasons. Apart of the low number of studies covering this topic, most of those available were carried out by using other methodological approaches, such as immunocytochemistry (Ariano et al., 1997; Defagot et al., 1997; Khan et al., 1998; Svingos et al., 2000; Wedzony et al., 2000; Rivera et al., 2003), radioligands (Primus et al., 1997; Defagot et al., 2000; De la Garza and Madras, 2000; Moreland et al., 2004), and real time polymerase chain reaction (RT-PCR) assays (Van Tol et al., 1991; Matsumoto et al., 1995, 1996; Suzuki et al., 1995; Araki et al., 2007) or *Drd4*-EGFP transgenic mice (Noaín et al., 2006), and there are no detailed studies using *in situ* hybridization techniques. Most of these reports are focused on restricted brain areas or nuclei (Meador-Woodruff et al., 1994, 1996, 1997; Lidow et al., 1998; Zhu et al., 2007). Even those studies analyzing wider regions of the brain omitted presenting results from particular areas or nuclei. In other cases the expression of the D4 receptor was analyzed only in specific developmental stages (zebrafish larvae; Boehmler et al., 2007). Finally, the overall set of reported results presents large discrepancies probably due to the use of non-specific antibodies or ligands (Kubikova et al., 2010; Rondou et al., 2010). However, studies in other vertebrates corroborate that the D4 receptor has a much more restricted expression pattern than the D2 receptor (Oak et al., 2000; Callier et al., 2003). This general conclusion is also true for the brain of the sea lamprey, where the D4 receptor exhibited a more restricted pattern than that of the D2 subtype (Pérez-Fernández et al., 2014b; present results).

#### Secondary Prosencephalon

In lampreys, weak D4 staining was observed in both the S and the RPa. The presence of a D4 dopamine receptor was also reported with different techniques in the S of other species analyzed, mostly at low levels (Surmeier et al., 1996; Ariano et al., 1997; Defagot and Antonelli, 1997; Defagot et al., 1997, 2000; Lidow and Goldman-Rakic, 1997; Tarazi et al., 1997, 1998a,b; Khan et al., 1998; Lidow et al., 1998; Mauger et al., 1998; De la Garza and Madras, 2000; Rivera et al., 2002, 2003; Callier et al., 2003; Moreland et al., 2004; Regard et al., 2008; Lacivita et al., 2010). Results obtained in humans are contradictory: although D4 presence could not be detected by various authors (Lahti et al., 1995, 1998; Meador-Woodruff et al., 1996, 1997; Primus et al., 1997), low levels were reported by using *in situ* hybridization (Van Tol et al., 1991; Stefanis et al., 1998), RT-PCR (Matsumoto et al., 1995, 1996; Mulcrone and Kerwin, 1997), and immunolabeling (Khan et al., 1998; Svingos et al., 2000). As reported in mammals by Oak et al. (2000), our results in the sea lamprey showed that the D4 expression level in the S is lower than that of the D2 receptor (present results; Pérez-Fernández et al., 2014b). The expression of a D4 receptor may explain why a few neurons responded to both D1 and D2 agonists in the lamprey S (Ericsson et al., 2013). It is likely that in lampreys, as in mammals (Surmeier et al., 1996; Lidow et al., 1998; Callier et al., 2003), there is some coexpression of D1 and D4 receptors. Given that the D4 receptor has been shown to regulate presynaptically the release of GABA in striatonigral neurons (Rivera et al., 2003), it seems that this mechanism might also be present in lampreys.

D4 expression was also detected in other prosencephalic areas of mammals, with the highest levels of expression in several cortical areas, including the hippocampus (O’Malley et al., 1992; Meador-Woodruff et al., 1994, 1996, 1997; Lahti et al., 1995, 1998; Ariano et al., 1997; Defagot et al., 1997; Lidow et al., 1998; De la Garza and Madras, 2000; Rivera et al., 2002; Callier et al., 2003; Noaín et al., 2006; de Almeida and Mengod, 2010). Following immunohistochemical and electron microscopy analysis it was shown that D4 receptors are present in GABAergic interneurons in both the cerebral cortex and hippocampus (Mrzljak et al., 1996), thus suggesting that they modulate the GABAergic transmission. Of interest, two of the few telencephalic regions observed to express the D4 receptor in the sea lamprey are the lateral and ventral pallial sectors, proposed to be the blueprint of the mammalian cortex (Ocaña et al., 2015). The general pattern we found in this species of lamprey, however, is more consistent with that reported for the avian forebrain (Kubikova et al., 2010), with very low levels of D4 expression. On the other hand, no D4 expression was reported in the S, the hippocampus, the nucleus accumbens, and the amygdala of *Drd4*-EGFP transgenic mice (Noaín et al., 2006).

Some labeled D4 cells were found in different portions of the lamprey hypothalamus. The presence of the D4 dopamine receptor in different hypothalamic areas was also reported in rats (O’Malley et al., 1992; Defagot et al., 1997; Primus et al., 1997; Khan et al., 1998; Moreland et al., 2004), mice (Suzuki et al., 1995; Huang et al., 2005), monkeys (De la Garza and Madras, 2000), and humans (Matsumoto et al., 1995; Mulcrone and Kerwin, 1997; Primus et al., 1997; reviewed in Callier et al., 2003). As an example of its function, it has been recently shown that this dopamine receptor plays a stimulatory role on feeding behavior regulation in rats (Tejas-Juárez et al., 2014).

#### Diencephalon

We observed scarce D4 labeling in the diencephalon, which was confined to the PE, the H, and the NMLF. In other vertebrates, including humans, a moderate D4 protein staining was reported in some thalamic areas by using immunocytochemistry (Mrzljak et al., 1996; Ariano et al., 1997; Khan et al., 1998), binding assays (Primus et al., 1997; De la Garza and Madras, 2000), RT-PCR (O’Malley et al., 1992 Matsumoto et al., 1995; Suzuki et al., 1995; Mulcrone and Kerwin, 1997), or northern blot (Van Tol et al., 1991) methods. In addition, some diencephalic expression was also reported in developing zebrafish embryos for the three D4 receptor genes (*drd4a*, *drd4b*, and *drd4c*) identified by Boehmler et al. (2007). On the other hand, thalamic D4 expression was not reported neither in the avian brain (Kubikova et al., 2010), nor in *Drd4*-EGFP transgenic mice (Noaín et al., 2006). From a functional point of view, some specific data have been reported concerning the regulation of the dopamine D4 receptor and its role in the rat lateral H, where it mediates inward currents (Root et al., 2015). In addition, it has been shown in rats that GABA release from pallidal terminals in the subthalamic nucleus, the thalamic reticular nucleus and the substantia nigra *pars reticulata* is inhibited by activation of presynaptic D4 receptors (Florán et al., 2004; Acosta-García et al., 2009; Gasca-Martínez et al., 2010; Govindaiah et al., 2010). In this respect, it should be noted that a putative pallidal region bearing GABAergic neurons and also projecting to the diencephalic locomotor region, the subthalamic region and the substantia nigra *pars reticulata* was identified by Stephenson-Jones et al. (2011) in the lamprey forebrain, which, according to these authors is located ventrolateral to the eminentia thalami (Stephenson-Jones et al., 2011). Therefore, we cannot rule out that a portion of the D4 positive cells located in the nSM (present results) might be included inside the limits of that putative pallidal region, thus being equivalent to the modulatory system present in the mammalian brain. In mammals, the dopaminergic innervation of the thalamic reticular nucleus arises from the substantia nigra *pars compacta* (Sánchez-González et al., 2005; Anaya-Martínez et al., 2006; García-Cabezas et al., 2007, 2009). Of interest, this projection could correspond with some of the TH-ir anterogradely labeled fibers innervating thalamic areas from the homolog of the mammalian substantia nigra *pars compact*a in lampreys (Pérez-Fernández et al., 2014b).

#### Mesencephalon

In the mesencephalic nIII of the sea lamprey we detected one of the strongest labeling for the D4 receptor. In addition, the local pair of large Müller cells (M3; reticular cells) was also labeled for this receptor, as well as some cells located in the M5 nucleus of Schöber. Although D4 expression was reported in some mesencephalic areas of other vertebrates, including the superficial layers of the superior colliculus, the inferior colliculus and the substantia nigra *pars reticulata*, (Van Tol et al., 1991; Matsumoto et al., 1995; Mrzljak et al., 1996; Defagot et al., 1997; Defagot and Antonelli, 1997; Mulcrone and Kerwin, 1997; Primus et al., 1997; Khan et al., 1998; De la Garza and Madras, 2000; Callier et al., 2003; Rivera et al., 2003; Kubikova et al., 2010), only one study reported positive cells in the nIII (De la Garza and Madras, 2000). A number of *drd4b* mRNA-expressing cells were also observed in the midbrain of developing zebrafish embryos (see Figure 4 in Boehmler et al., 2007); however, they were not ascribed to specific nuclei. In addition, no D4 positive cells were described in the mesencephalon of *Drd4*-EGFP transgenic mice (Noaín et al., 2006).

#### Rhombencephalon

The rhombencephalon is the part of the brain exhibiting the highest levels of D4 expression in the sea lamprey. In general, our results disagree with the relatively low D4 expression reported so far in other vertebrates, which, at least in part, is due to the absence of detailed studies (Van Tol et al., 1991; Matsumoto et al., 1996; Noaín et al., 2006; Boehmler et al., 2007; Kubikova et al., 2010). However, a high number of D4 positive cells was reported in the mammalian cerebellum (Matsumoto et al., 1995, 1996; Suzuki et al., 1995; Ariano et al., 1997; Defagot and Antonelli, 1997; Defagot et al., 1997; Mulcrone and Kerwin, 1997; Khan et al., 1998; De la Garza and Madras, 2000; Moreland et al., 2004; Lacivita et al., 2010), with the exception of the study carried out by Noaín et al. (2006), as well as in the avian cerebellum (Kubikova et al., 2010). However, lampreys do not possess a “true” cerebellum (see Pombal and Megías, 2011), which precludes comparison.

Concerning other rhombencephalic areas, data are even more fragmentary, with little agreement among the few available studies. On one hand, some D4 expression was reported in the rhombencephalon of the teleostean (Boehmler et al., 2007), avian (Kubikova et al., 2010), and human (Van Tol et al., 1991; Matsumoto et al., 1996) brains, but the authors did not describe in which specific areas or nuclei it was found. By using autoradiography, De la Garza and Madras (2000) described the presence of the D4 receptor associated to the abducens, gigantocellular, vestibular, cochlear, and inferior olivary nuclei in monkeys, with the highest binding in the nVI (De la Garza and Madras, 2000). Other authors detected some D4 positive cells in the locus coeruleus, the parabrachial nuclei and the raphe nucleus of rats (Defagot et al., 1997; Primus et al., 1997; Noaín et al., 2006) or the dorsal vagal complex of humans (Hyde et al., 1996). Moreover, in the work carried out by Callier et al. (2003), no D4 positive cells were reported in any rhombencephalic region or nuclei. Finally, although Khan et al. (1998) did not report any D4 expression in the rhombencephalon of rats and humans, it is not clear whether the authors did not find any D4 positive cell in this part of the brain or if this feature was not studied there. Bearing in mind this scenario, there is little agreement between our results in the sea lamprey and those available for other vertebrates.

The most salient finding in the rhombencephalon of the sea lamprey is the apparent correspondence between the D4 labeled cell populations reported herein and those immunoreactive for choline acetyltransferase (ChAT; Pombal et al., 2001). This is clear for the labeled cell populations located in the isthmic region, the somatomotor and visceromotor nuclei, and even the few and small cells observed medially to the nIX and nX. Therefore, a colocalization of the D4 receptor and ChAT is very likely in all these cell populations, and thus may represent a neuroanatomical substrate involved in mediating dopaminergic influences on acetylcholine release. To our knowledge, there are no similar results in other species of vertebrates and more studies are necessary to examine whether there is any functional relationship between these two neurotransmitters. Taken together, our findings as well as those reported for the D2 receptor by Pérez-Fernández et al. (2014b) suggest that the D2 family of receptors represents an important component of brainstem mechanisms regulating visceral functions.

#### Spinal Cord

We detected intensely labeled D4 cells in the lamprey spinal cord. D4 receptor expression was previously found in mouse spinal cord by using RT-PCR and *in situ* hybridization (Zhu et al., 2007). There is indeed a study reporting significant levels of D4 mRNA in the human spinal cord by using the RT-PCR method (Matsumoto et al., 1996). In addition, two (drd4a and drd4c) of the three dopamine receptors identified in zebrafish were first detected in the spinal cord at 24 h post fertilization by using whole mount *in situ* hybridization (Boehmler et al., 2007). These authors also studied the hypoactivity effect of clozapine, a selective D4 receptor antagonist, on swimming behavior of zebrafish larvae, and they concluded that this effect is consistent with the view that clozapine is acting through D4 rather than D2 or D3 receptors.

As observed in other vertebrates, our results show that the general D4 receptor expression pattern in lamprey is much more restricted than that of the D2 receptor, whose expression was also reported in areas such as the OBs, dorsomedial telencephalic neuropil, septum, globus pallidus, MAM, nucleus of the posterior tuberculum, dorsal and ventral thalamus, pretectum, OT, TS, mesencephalic tegmentum, substantia nigra *pars reticulata*, IP, ndV, DCN, or around the SMC (present results; Pérez-Fernández et al., 2014b). In addition, the expression of D4 appears to be conserved throughout vertebrate evolution in areas such as the S (Surmeier et al., 1996; Ariano et al., 1997; Defagot and Antonelli, 1997; Defagot et al., 1997, 2000; Lidow and Goldman-Rakic, 1997; Tarazi et al., 1997, 1998a,b; Khan et al., 1998; Lidow et al., 1998; Mauger et al., 1998; De la Garza and Madras, 2000; Rivera et al., 2002, 2003; Callier et al., 2003; Moreland et al., 2004; Regard et al., 2008; Lacivita et al., 2010). There are other areas where the D4 expression might also be conserved in the different animal groups analyzed. However, the lack of detailed expression maps together with potential problems related with the sensitivity and the variety of techniques used (see Introduction) makes it difficult to compare available data (and therefore to establish putative homologies). Even so, our *in situ* hybridization studies in lampreys show significant overlap with previous results on the D4 expression within specific areas of the mammalian brain.

### Comparative Analysis with the D2 Receptor Expression

As stated above, the D4 receptor expression pattern is much more restricted than that of the D2 receptor (present results; Pérez-Fernández et al., 2014b), but both receptors can be found co-expressed in several brain regions. In the prosencephalon, D2 and D4 expression can be found in the S, LP, VP, RPa, nTPOC, TN, TM, H, and the NMLF. Despite sharing areas of expression, it is remarkable that, at least in some regions, the D4 receptor expression is likely to be complementary to that of the D2 receptor. This is evident in the rhombencephalon, where D2 expression is only found in small cells intermingled with the motoneurons of the different motor nuclei, which however express the D4 receptor (Pérez-Fernández et al., 2014b; present results), but also in the mesencephalon, where D4 expression is found in the motoneurons of the nIII, as well as in the M5 nucleus of Schöber and the giant Müller cells, which are devoid of D2 expression (Pérez-Fernández et al., 2014b; present results). In the telencephalon it is difficult to state whether D2 and D4 positive cells are different populations without carrying out double assays. Anyway, our results suggest that the mechanism by which both subtypes were conserved after duplication may be a subfunctionalization process (Conrad and Antonarakis, 2007).

### Comparative Analysis with TH Immunoreactivity

Several studies have been carried out in larval and adult specimens of different lamprey species analyzing tyrosine hydroxylase (TH) and dopamine (DA) immunoreactivities (Pierre et al., 1994, 1997; Pombal et al., 1997; Pierre-Simons et al., 2002; Abalo et al., 2005). TH is a key enzyme in dopamine synthesis that has been shown to be a reliable marker to detect catecholaminergic transmitters in the lamprey brain (Pierre et al., 1997; Pombal et al., 1997). In the lamprey prosencephalon, D4 positive cells were found in the S, which receives strong dopaminergic innervation from the nucleus of the posterior tuberculum, proposed to be the homolog of the substantia nigra *pars compacta*/ventral tegmental area of mammals (Pombal et al., 1997; Stephenson-Jones et al., 2011; Pérez-Fernández et al., 2014b). In the preoptic area, D4 positive cells were found in the RPa, where TH- and DA-immunoreactive (ir) cells were also shown (Pierre et al., 1994, 1997; Pombal et al., 1997; Pierre-Simons et al., 2002; Abalo et al., 2005); therefore, some of these cells are likely to have autoreceptors. In other prosencephalic regions, a rich dopaminergic innervation was reported in all the areas shown to express the D4 receptor, excepting the H. This is the case of the LP, VP, nTPOC, TN, TM, and the NMLF. In the H, where some D4 receptor expressing cells are present, only a few TH- and DA-ir fibers were reported (Pierre et al., 1997; Pombal et al., 1997; Abalo et al., 2005).

Concerning mesencephalic and rhombencephalic areas, abundant TH- and DA-ir fibers were reported in the area of distribution of the dendrites of the motoneurons of the different motor nuclei (Pierre et al., 1994, 1997; Pombal et al., 1997; Pierre-Simons et al., 2002; Abalo et al., 2005), which show a strong D4 receptor expression. In the isthmic region, where numerous D4 positive cells are present, abundant TH- and DA-ir fibers and a few cells were also reported. This is also the case of the spinal cord, where abundant dopaminergic fibers are present in the motor column of the gray substance, where some D4-expressing motoneurons are observed (present results; Pombal et al., 1997).

### Evolutionary History of the D2 Class Receptors

Controversy still exists concerning the moment of divergence of lampreys regarding the two WGDs proposed by the 2R hypothesis. Lately, many authors accept that such divergence occurred between the two WGDs proposed by the 2R hypothesis (see Kasahara, 2007 and Osório and Rétaux, 2008). On the other hand, after sequencing the whole genome of *P. marinus*, it was proposed that lampreys diverged after the two WGDs (Smith et al., 2013); however, these results are not fully conclusive and the divergence of lampreys remains an open question. We have previously found a repertoire of NPY receptors consistent with lampreys diverging between the two WGDs (Pérez-Fernández et al., 2013, 2014a). The existence of two members within the D2 class of dopamine receptors in these animals also supports this hypothesis (Robertson et al., 2012; Pérez-Fernández et al., 2014b; present work).

**FIGURE 9:**
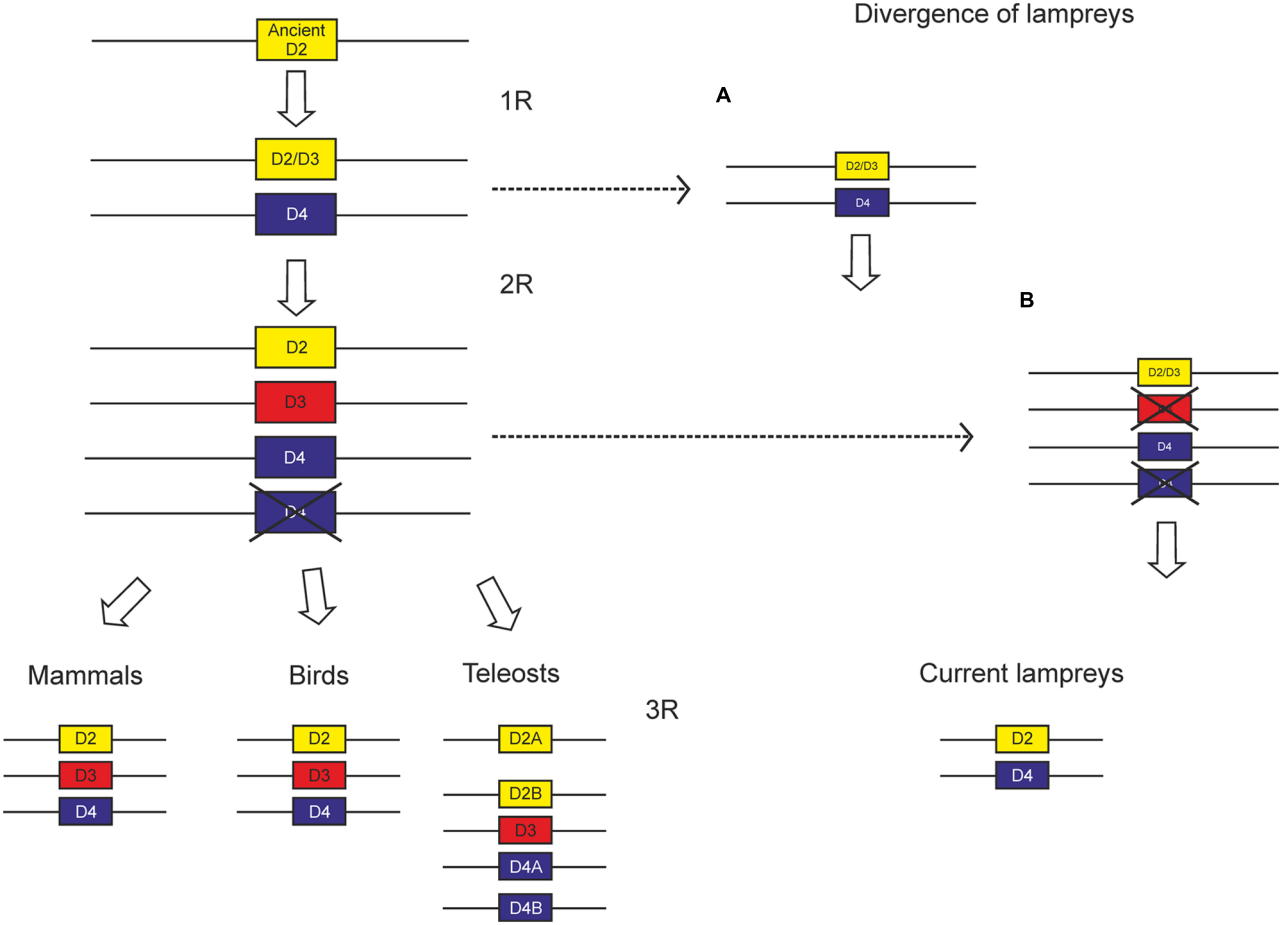
**Scheme proposed to explain the evolution of D2 class receptors based on the existing data.** Since the divergence of lampreys is not yet clear, we propose two alternative scenarios, the first one **(A)** with lampreys diverging between the two WGDs proposed by the 2R hypothesis, and the other one **(B)** with lampreys diverging after the two WGDs.

So far, no suggestions have been made concerning the evolution of the D2 class of dopamine receptors. Since it is clear that lampreys diverged after the first WGD, there are two putative scenarios for the evolution of the D2 class receptors, depending on the moment of divergence of lampreys (**Figure 9**). It is likely that two members arose from an ancient D2 class receptor by means of the first WGD, which were conserved until the second WGD. According to the repertoire of D2 class receptors, with a D2 and a D4 receptor, it is likely that lampreys diverged before this second WGD. This scenario would lead to the presence of four D2 class receptors in the second WGD, but most likely one duplicate was lost before the gnathostomes split, since only three members have been found in all groups of gnathostomes analyzed. The only exception is teleosts, which show additional members due to the third round of WGD occurred in this group (3R, see Introduction).

## Conclusion

In lampreys, the D4 receptor is expressed in several encephalic areas such as striatum, lateral and ventral pallial sectors, hypothalamus, habenula, and several motor nuclei. These results reinforce the idea of a complex dopaminergic system in these animals (Pérez-Fernández et al., 2014b), at the dawn of vertebrate evolution. Our data are relevant given the scarce number of studies analyzing the D4 receptor expression profile, even in mammalian species.

## Author Contributions

All authors had full access to all the data in the study and take responsibility for the integrity of the data and the accuracy of the data analysis. JPF, MM, and MAP conceived and designed the study. JPF performed the experiments. JPF and MAP were the primary contributors to the data analysis. JPF, MM, and MAP prepared the figures and wrote the paper. All authors approved the article.

## Conflict of Interest Statement

The authors declare that the research was conducted in the absence of any commercial or financial relationships that could be construed as a potential conflict of interest.

## Footnotes

1 http://genome-euro.ucsc.edu/index.html

2 http://www.ncbi.nlm.nih.gov/genbank

3 http://www.ensembl.org/Petromyzon_marinus/Info/Index

4 http://blast.ncbi.nlm.nih.gov/Blast.cgi

5 http://smart.embl-heidelberg.de

6 http://octopus.cbr.su.se/

7 http://genes.mit.edu/GENSCAN.html

## Abbreviations

AH: anterior hypothalamus
ARN: anterior rhombencephalic reticular nucleus
cc: central canal
ch: optic chiasm
cp: cerebellar plate
CPa: caudal paraventricular nucleus
DCN: dorsal column nucleus
dI: dorsal isthmic nucleus
GS: gray substance of the spinal cord
H: habenula
IP: interpeduncular nucleus
LP: lateral pallium
M3: Müller cell 3
M5: mesencephalic retinopetal nucleus of Schöber
MAM: mamillary region
MPO: medial preoptic nucleus
MRN: medial rhombencephalic reticular nucleus
ndV: sensory nucleus of the descending trigeminal tract
nIII: oculomotor nucleus
nIV: trochlear motor nucleus
nIX: glossopharyngeal motor nucleus
NMLF: nucleus of the medial longitudinal fasciculus
NRM: mesencephalic reticular nucleus
nSM: nucleus of the stria medullaris
nSO: spino-occipital motor nucleus
NST: nucleus of the solitary tract
nTPOC: nucleus of the tract of the postoptic commissure
nV: trigeminal motor nucleus
nVI: abducens motor nucleus
nVII: facial motor nucleus
nXc: caudal portion of the vagal motor nucleus
nXr: rostral portion of the vagal motor nucleus
OB: olfactory bulb
og: glomerular layer of the olfactory bulb
OLA: octavolateral area
OT: optic tectum
PE: prethalamic eminence
PEA: pallial extended amygdala
PRN: posterior rhombencephalic reticular nucleus
PTh: prethalamus
PVO: periventricular hypothalamic organ
RPa: rostral paraventricular nucleus
S: striatum
SMC: spinal motor column
Th: thalamus
TM: tuberomamillary nucleus
TN: tuberal nucleus
TS: torus semicircularis
VP: ventral pallium
